# Pulmonary Vein Stenting With Hemodynamics and Intravascular Ultrasound Guidance

**DOI:** 10.1016/j.jaccas.2025.104408

**Published:** 2025-07-30

**Authors:** Rasha Al-Bawardy, Wail Alkashkari, Mohammed Althobaiti, Abdulhalim Kinsara, Amin Zagzoog, Atif AlQubbany, Abdullah Alsaiedi

**Affiliations:** a King Faisal Cardiac Center, King Abdulaziz Medical City, Ministry of National Guard Health Affairs, Jeddah, Saudi Arabia; b King Abdullah International Medical Research Center, Jeddah, Saudi Arabia; c College of Medicine, King Saud Bin Abdulaziz University for Health Sciences, Jeddah, Saudi Arabia; d Department of Radiology, King Abdulaziz Medical City, Ministry of National Guard Health Affairs, Jeddah, Saudi Arabia

**Keywords:** hemodynamics, IVUS guidance, pulmonary vein stenosis, pulmonary vein stenting

## Abstract

**Background:**

Pulmonary venous stenosis (PVS) is a rare but serious complication of atrial fibrillation pulmonary vein isolation, often diagnosed late because of vague symptoms. Stenting is a potential treatment in select cases.

**Case Summary:**

A 49-year-old woman with atrial fibrillation underwent pulmonary vein isolation a year ago and presented multiple times with dyspnea and hemoptysis. She was eventually diagnosed with PVS of the left inferior pulmonary vein (LIPV) and occlusion of the left superior pulmonary vein. Invasive hemodynamics showed segmental pulmonary hypertension, with an elevated pulmonary capillary wedge pressure (PCWP) of 53 mm Hg in the affected lung vs 22 mm Hg in the unaffected side. She underwent LIPV stenting using intravascular ultrasound guidance and a drug-eluting stent. After stenting, no gradient remained across the LIPV, and the left PCWP dropped to 26 mm Hg. Hemoptysis resolved.

**Discussion:**

This case highlights segmental worse pulmonary hypertension and PCWP due to PVS, effectively treated with intravascular ultrasound–guided stenting.

## Graphical Abstract

**Figure:**
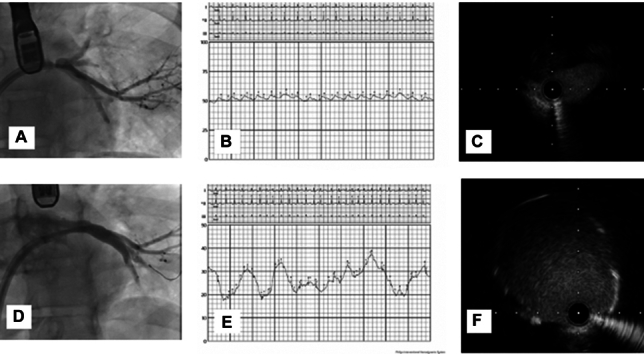

**Visual Summary:**
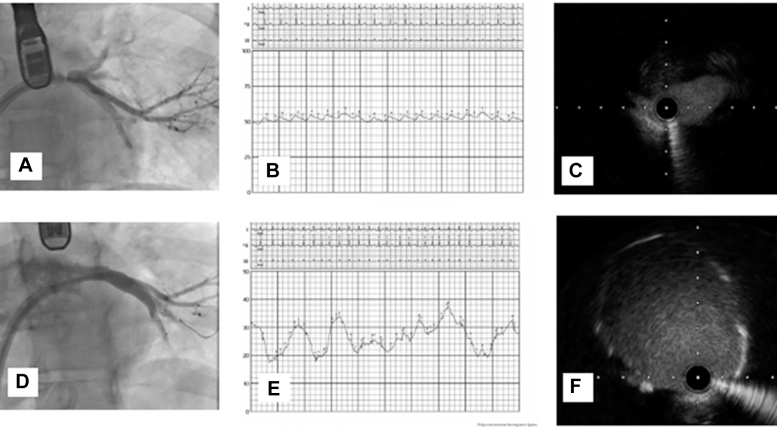
Prestenting and Poststenting Prestenting: (A) angiography with a severe LIPV lesion, (B) L PCWP = 53 mm Hg, and (C) IVUS at the site of the lesion. Poststenting: (D) angiography with resolution of the LIPV lesion, (E) L PCWP = 26 mm Hg, and (F) IVUS with a well-expanded, well-apposed stent. IVUS = intravascular ultrasound; L PCWP = left pulmonary capillary wedge pressure.

## History of Presentation

A 49-year-old woman with atrial fibrillation (AFib) who underwent pulmonary vein isolation (PVI) a year ago presented with progressive dyspnea on exertion (DOE) and hemoptysis. She remained in normal sinus rhythm after the procedure with no known recurrence. Dyspnea began approximately 2 months after the procedure and gradually worsened, followed by hemoptysis. She had multiple emergency department visits due to increasing frequency of her hemoptysis. Pulmonary embolism was initially suspected but ruled out. She was hemodynamically stable, with unremarkable physical examination findings, including normal cardiac and pulmonary examination findings.

## Past Medical History

Her primary medical history includes AFib for which she underwent PVI a year ago.

## Differential Diagnosis

Initial symptoms of DOE before hemoptysis suggested a broad differential diagnosis, including both cardiac and pulmonary etiologies. Cardiac causes included heart failure, valvular disease, coronary artery disease, and recurrent arrhythmia—although no palpitations were reported and her symptoms differed from her prior AFib episodes. Pulmonary considerations included pulmonary embolism (particularly given her hemoptysis), pulmonary vein stenosis (considering her PVI history), and lung masses.

## Investigations

Because of her vague symptoms, she underwent multiple evaluations during her emergency department visits. Electrocardiography showed normal sinus rhythm with no significant abnormalities. Transthoracic echocardiography revealed normal left and right ventricular size and systolic function, normal ejection fraction, and no significant valvular disease. Chest radiograph findings were unremarkable. After presentation with hemoptysis, computed tomography (CT) angiography of the chest ruled out pulmonary embolism. She was referred to pulmonology for bronchoscopy and to cardiology for the evaluation of DOE. Further review of the CT pulmonary embolism scan raised suspicion of pulmonary vein stenosis (PVS). A dedicated CT confirmed occlusion of the left superior pulmonary vein (LSPV) and severe stenosis of the left inferior pulmonary vein (LIPV). A V/Q scan demonstrated a perfusion mismatch, primarily in the left lower lobe.

## Management

**Figure 1:**
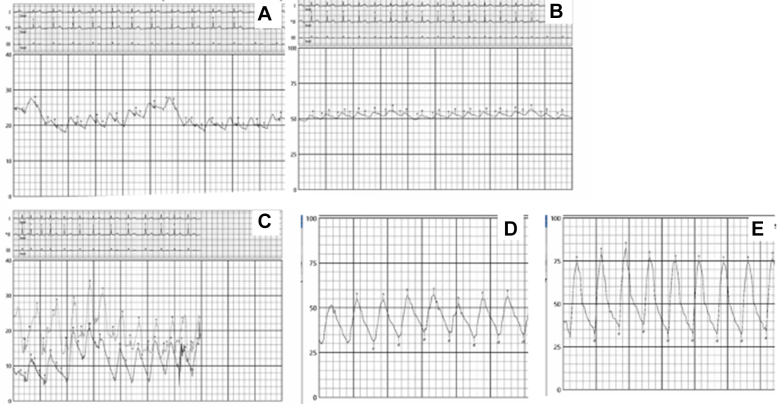
Detailed Invasive Hemodynamics Assessment Performed Before Pulmonary Vein Stenting (A) PCWP from the right (unaffected) side with a mean of 22 mm Hg. (B) PCWP from the left (affected) side with a mean of 53 mm Hg. (C) Left atrial pressure of 12 mm Hg and left inferior pulmonary vein 20 to 22 mm Hg with a gradient of 8 to 10 mm Hg. (D) Right PA pressure = 56/35 mm Hg (mean 45 mm Hg). (E) Left PA pressure = 76/34 mm Hg (mean 53 mm Hg). PA = pulmonary artery; PCWP = pulmonary capillary wedge pressure.

**Figure 2:**
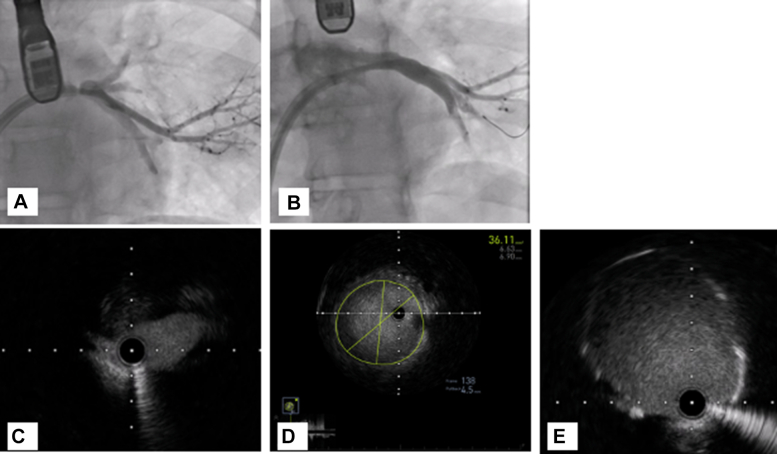
Angiography and IVUS Before and After Stenting (A) Selective LIPV angiography before stenting. (B) Selective LIPV angiography after stenting. (C) IVUS showing the lesion at the proximal LIPV with no calcifications. (D) Distal reference on IVUS. (E) Poststenting IVUS at the lesion site with well-expanded and well-apposed stent. IVUS = intravascular ultrasound; LIPV = left inferior pulmonary vein.

**Figure 3:**
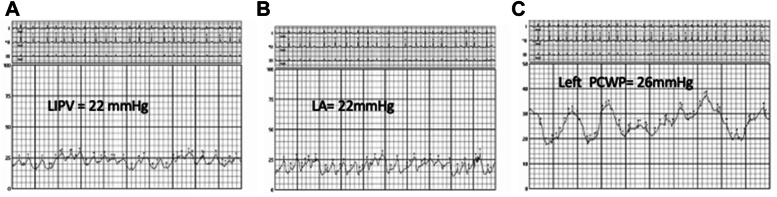
Hemodynamics After Stenting (A) LIPV pressure = 22 mm Hg. (B) Left atrial pressure = 22 mm Hg with no gradient on pullback from the LIPV. (C) Left PCWP = 26 mm Hg (was 53 mm Hg). LA = left atrium; LIPV = left inferior pulmonary vein; PCWP = pulmonary capillary wedge pressure.

She underwent invasive hemodynamic assessment before percutaneous pulmonary vein stenting (Figure 1). Hemodynamics via right femoral vein access and a pulmonary artery (PA) catheter revealed a left PA pressure of 76/34 mm Hg (mean 53 mm Hg) and a right PA pressure of 56/35 mm Hg (mean 45 mm Hg). The pulmonary capillary wedge pressure (PCWP) was 53 mm Hg on the left and 22 mm Hg on the right. After trans-septal puncture and engagement of the LIPV, an 8 to 10 mm Hg gradient was noted between the LIPV and left atrium. Transesophageal echocardiography–guided trans-septal puncture was performed. An Agilis NxT (Abbott) catheter was used to steer the multipurpose catheter, and the LIPV was engaged. A Terumo wire was advanced, followed by exchange to a 0.014 inch Ironman wire. Intravascular ultrasound (IVUS) identified a short-segment lesion with a distal reference diameter of around 6 mm (Figure 2). The lesion was predilated using a 4.0 × 10 mm WOLVERINE cutting balloon (Boston Scientific) and stented with a 5.0 × 12 mm XIENCE DES (Abbott), followed by postdilation with a 6 × 12 mm noncompliant balloon. Final IVUS showed a well-expanded, well-apposed stent (Figure 2). After intervention, there was no gradient across the LIPV into the left atrium, and the left PCWP dropped from 53 to 26 mm Hg, matching the right side. The left PA pressure decreased to 52/42 mm Hg (mean 44 mm Hg), also comparable to the right (Figure 3). The LSPV was not treated because of total occlusion and favorable hemodynamic improvement.

## Outcomes and Follow-Up

The patient experienced complete resolution of hemoptysis and DOE, with no symptom recurrence over 8 months. She was initially managed on anticoagulation and a single antiplatelet agent. After 5 months, antiplatelet therapy was discontinued, and she continued on apixaban alone due to recurrent AFib episodes observed during and after the procedure.

## Discussion

Iatrogenic PVS is not uncommon after PVI, with imaging at 6 months after the procedure detecting stenosis in 30% to 40% of patients.^1^ However, most cases are mild and asymptomatic, with only 0.5% to 4% being severe.^1^ Despite advances in ablation techniques aimed at reducing this complication, there are some unclear patient factors that may still contribute to having severe symptomatic PVS.

Asymptomatic cases can be managed conservatively. However, symptomatic cases are often misdiagnosed initially because of overlapping symptoms with many other conditions, requiring a high index of suspicion. Although invasive hemodynamic assessment is rarely used, it has been described in the literature to reveal segmental pulmonary hypertension—elevated PA pressure and PCWP in the affected lung compared with the unaffected lung. An alternative hemodynamic marker not assessed in this case is a PCWP-left ventricle diastolic gradient (without a left atrium-left ventricle gradient as seen in mitral stenosis), which has diagnostic value in PVS.

In this case, successful stenting equalized left and right PCWP values and resolved segmental pulmonary hypertension, which likely contributed to symptom resolution. Therefore, the remaining occluded LSPV was not intervened on. In addition, the use of IVUS in pulmonary vein stenting is rarely reported, typically in congenital cases.^2^, ^3^, ^4^ Here, IVUS facilitated in lesion assessment and ensured adequate stent expansion. Although its role in predicting long-term outcomes or restenosis is unclear, its use may help optimize immediate results and warrants further study. The role of hemodynamics and IVUS guidance in predicting outcomes and future recurrence is limited but is worth investigating as it might help in reducing recurrence and in-stent restenosis.

## Conclusions

Pulmonary vein stenosis is a rare but important complication of PVI that is often diagnosed late because of nonspecific symptoms and initial evaluation by noncardiologists. Invasive hemodynamics can reveal segmental pulmonary hypertension and higher PCWP in the affected side compared with the unaffected side, and IVUS-guided stenting offers an effective treatment strategy with potential for favorable outcomes.

## Funding Support and Author Disclosures

Dr Al-Bawardy reported consulting/honoraria fees from Amgen, Boston Scientific, Medtronic, and Terumo. All other authors have reported that they have no relationships relevant to the contents of this paper to disclose.

**Take-Home Messages**

• Pulmonary vein stenosis is a rare complication of pulmonary vein isolation and requires a high index of suspicion for timely diagnosis.
• Invasive hemodynamics can play a role in diagnosis with segmental pulmonary hypertension and higher pulmonary capillary wedge pressure in the affected lung compared with the unaffected lung.
• In our case, invasive hemodynamics helped in guiding management, with stenting only the left inferior pulmonary vein and leaving the totally occluded left superior pulmonary vein untreated given favorable hemodynamics outcomes after left inferior pulmonary vein stenting.
• The use of intravascular ultrasound can help in pulmonary vein stent optimization and immediate procedural success, but its role in predicting restenosis remains unclear.
